# Broadening the horizon – level 2.5 of the HUPO-PSI format for molecular interactions

**DOI:** 10.1186/1741-7007-5-44

**Published:** 2007-10-09

**Authors:** Samuel Kerrien, Sandra Orchard, Luisa Montecchi-Palazzi, Bruno Aranda, Antony F Quinn, Nisha Vinod, Gary D Bader, Ioannis Xenarios, Jérôme Wojcik, David Sherman, Mike Tyers, John J Salama, Susan Moore, Arnaud Ceol, Andrew Chatr-aryamontri, Matthias Oesterheld, Volker Stümpflen, Lukasz Salwinski, Jason Nerothin, Ethan Cerami, Michael E Cusick, Marc Vidal, Michael Gilson, John Armstrong, Peter Woollard, Christopher Hogue, David Eisenberg, Gianni Cesareni, Rolf Apweiler, Henning Hermjakob

**Affiliations:** 1 European Bioinformatics Institute, Wellcome Trust Genome Campus, Hinxton, Cambridge, UK; 2 Banting & Best Department of Medical Research and Terrence Donnelly Centre for Cellular & Biomolecular Research, University of Toronto, 160 College Street, Toronto, Ontario, Canada; 3 Samuel Lunenfeld Research Institute, Mount Sinai Hospital, 600 University Avenue, Toronto, Ontario, Canada; 4 Merck Serono, 9 chemin des Mines, 1211 Geneva, Switzerland; 5 Laboratoire Bordelais de Recherche en Informatique, ENSI Électronique, Informatique et Radiocomm. de Bordeaux, France; 6 The Blueprint Initiative of Mount Sinai Hospital, 600 University Avenue, Toronto, ON, M5G 1X5, Canada; 7 National University of Singapore, Office of Life Sciences (OLS), Centre for Life Sciences, Singapore; 8 Department of Biology, University of Rome Tor Vergata, Via della Ricerca Scientifica, Rome, Italy; 9 Institute for Bioinformatics, GSF – National Research Center for Environment and Health, Neuherberg, Germany; 10 UCLA-DOE Institute for Genomics & Proteomics, UCLA, LA, USA; 11 Computational Biology Center, Memorial Sloan-Kettering Cancer Center 1275 York Avenue, Box 460, New York, NY, USA; 12 Center for Cancer Systems Biology (CCSB) and Department of Cancer, Biology, Dana-Farber Cancer Institute, and Department of Genetics, Harvard Medical School, Boston, MA, USA; 13 Center for Advanced Research in Biotechnology, University of Maryland Biotechnology Institute, Rockville, MD, USA; 14 Glaxo Smithkline Medicines Research Centre, Gunnels Wood Road, Stevenage, Herts, UK; 15 Dept. of Biochemistry, University of Toronto, Toronto, Ontario, Canada

## Abstract

**Background:**

Molecular interaction Information is a key resource in modern biomedical research. Publicly available data have previously been provided in a broad array of diverse formats, making access to this very difficult. The publication and wide implementation of the Human Proteome Organisation Proteomics Standards Initiative Molecular Interactions (HUPO PSI-MI) format in 2004 was a major step towards the establishment of a single, unified format by which molecular interactions should be presented, but focused purely on protein-protein interactions.

**Results:**

The HUPO-PSI has further developed the PSI-MI XML schema to enable the description of interactions between a wider range of molecular types, for example nucleic acids, chemical entities, and molecular complexes. Extensive details about each supported molecular interaction can now be captured, including the biological role of each molecule within that interaction, detailed description of interacting domains, and the kinetic parameters of the interaction. The format is supported by data management and analysis tools and has been adopted by major interaction data providers. Additionally, a simpler, tab-delimited format MITAB2.5 has been developed for the benefit of users who require only minimal information in an easy to access configuration.

**Conclusion:**

The PSI-MI XML2.5 and MITAB2.5 formats have been jointly developed by interaction data producers and providers from both the academic and commercial sector, and are already widely implemented and well supported by an active development community. PSI-MI XML2.5 enables the description of highly detailed molecular interaction data and facilitates data exchange between databases and users without loss of information. MITAB2.5 is a simpler format appropriate for fast Perl parsing or loading into Microsoft Excel.

## Background

Molecular interaction data is a key resource in modern biomedical research, and molecular interaction datasets are currently generated on a large scale, demonstrating from one to tens of thousands of interactions per experiment. These interaction data sets are represented in many different forms, from simple pairs of protein names to detailed textual descriptions, and are collected in various databases, each with their own database schema. In 2004, the HUPO Proteomics Standards Initiative developed and published the PSI-MI XML1.0 format for molecular interactions [1] as a community format for the exchange of protein interaction data. This format had been jointly developed by major producers of protein interaction data and by data providers, among them BIND [2], DIP [3], IntAct [4], MINT [5], MIPS [6] and Hybrigenics [7]. The PSI-MI XML1.0 format was widely implemented and supported by both software tool development and data providers.

The PSI-MI format was explicitly intended to develop in an incremental fashion. As a first step, version 1.0 focused exclusively on protein interactions, and provided only very limited support for quantitative parameters, such as kinetic measurements. As a direct result of requests from users, database groups and data providers, the HUPO-PSI work group for molecular interactions has significantly extended the capabilities of the original PSI-MI format resulting in version 2.5, presented here. The main features that have been added to the format broaden the range of interactor types, extend the descriptions that can be made of both experimental conditions and features on participating molecules, and add kinetic and modelled interaction parameters.

## Results

We have developed version 2.5 of the PSI-MI XML schema for molecular interactions, extended the associated controlled vocabularies and updated the tools supporting the format. In the following pages, we will provide a general description of the PSI-MI XML2.5 format, pointing out the major changes and additions with respect to level 1.0. A complete, detailed documentation of the PSI-MI XML2.5 schema is available at [8].

### The XML schema

**Figure 1:**
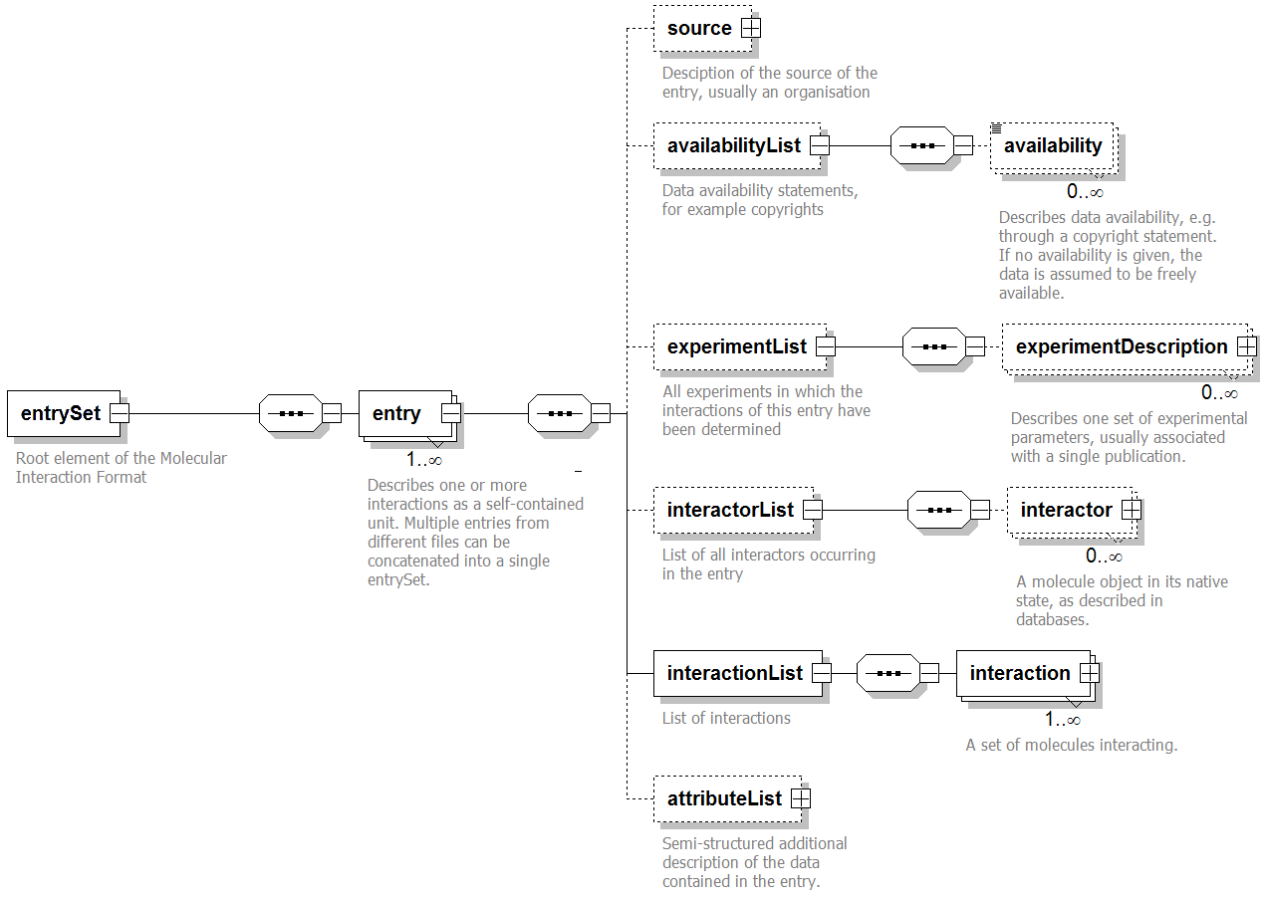
**Graphical representation of the PSI-MI XML2.5 format**. Some elements have been collapsed for clarity (indicated by a '+' in a rectangular box).

The root element of PSI-MI XML2.5 is the *entrySet*. It contains one or more *entry *elements. An *entry *is the core element of PSI-MI XML2.5, describing one or more interactions with all associated data as a self-contained unit (Figure 1). Thus, several PSI-MI XML2.5 files can easily be merged by inserting all *entry *elements into a single *entrySet*. The *entry *contains a source element describing the origin of the data in the *entry*, usually an organisation such as a database provider. The following three elements, *availabilityList*, *experimentList*, and *interactorList*, are container elements for repetitive elements of interactions. The next element, *interactionList*, contains all interactions described in the *entry*.

The PSI-MI XML2.5 schema allows two different representations of interactions – compact and expanded. In the compact form, the repetitive elements of a larger set of interactions, namely copyright statements, experiment descriptions, and interactors (proteins, small molecules, etc), are only described once, in the respective list elements. The interaction elements in the *interactionList *then only refer to the previously defined interactors and experiments through elements of type *idRef*, similar to the normalised representation in a relational database. The compact form is more suitable for larger datasets, where, for example, one protein would be referred to by multiple interaction elements.

Alternatively, the expanded form of the PSI-MI XML2.5 schema can be used. In this form, the *availabilityList*, *experimentList*, and *interactorList *are not used, and all *experiment*, *interaction*, and *availability *elements are provided within the relevant *interaction *elements, potentially repeating the same description multiple times throughout an *entry *in a manner comparable to a renormalized relational database. The expanded form is more suitable for smaller datasets, as it groups all related data closely together. The format presents further advantages in that interactions are now self-contained units thus rendering streaming of data much simpler and parsing more efficient. The compact and expanded form can be automatically inter-converted by XSLT scripts as described in the 'tools' section.

An *experimentDescription *provides a set of experimental parameters, which is usually associated with a single publication. However, a single publication will result in more than one *experimentDescription *element in PSI-MI XML2.5 representation, if the authors have used different technologies to confirm interactions. In the PSI-MI group, there is broad agreement that the experimental technologies to demonstrate a particular interaction are a key element in interpreting molecular interaction data. However, a full description of experimental detail, as specified in recent journal guidelines [9,10], is beyond the scope of the current PSI-MI XML2.5 schema. The *experimentDescription *focuses on a classification of experiments according to the methods used. Descriptions of experimental details, such as sample preparation or instrumentation used, will be modelled in detail in a future version, probably in the context of the maturing Functional Genomics Experiment (FuGE) [11] data model. As with PSI-MI XML1.0, the three method classifiers on experiment level are the *interactionDetectionMethod*, the *participantIdentificationMethod *and the *featureDetectionMethod*. Further key elements of the *experimentDescription *are the *bibref *bibliographic reference to the data source, and the *hostOrganismList *describing the environment in which the experiment has been performed. The latter is described using the NCBI taxonomy identifiers, with the addition of -1 to indicate '*in vitro*', -2 to indicate 'chemical synthesis', -3 to indicate 'unknown', -4 to indicate '*in vivo*' and -5 to indicate '*in silico*'.

**Figure 2:**
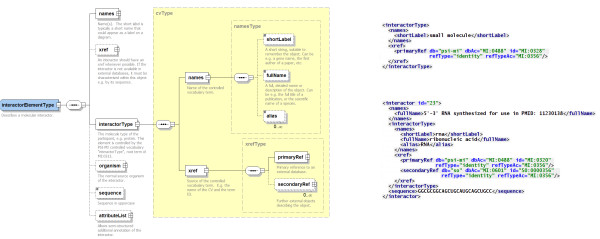
Use of *interactorType *within the PSI-MI XML2.5 schema to describe the molecular class of two participating molecules – a small molecule interactor and a RNA – using the appropriate controlled vocabulary term

**Figure 3:**
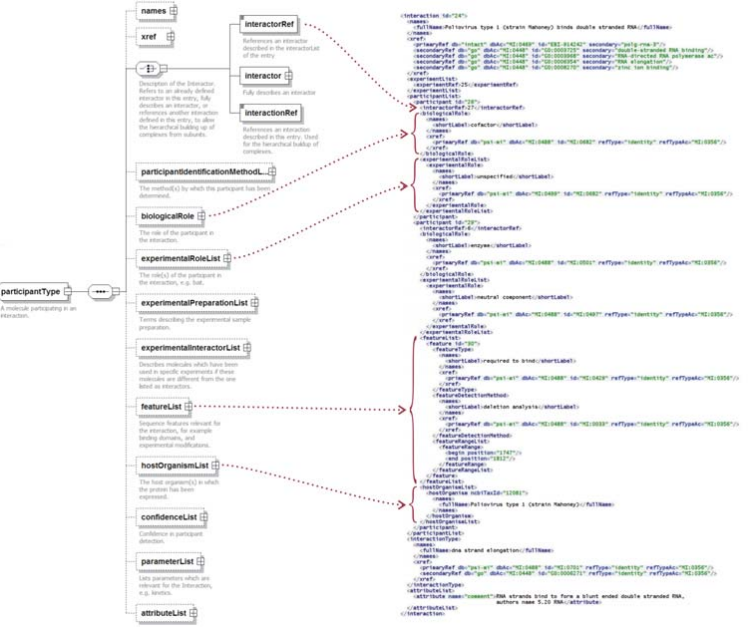
Graphical representation of section of the PSI-MI XML2.5 schema describing *participant *with the corresponding lines of XML containing sample data from [26]

**Figure 4:**
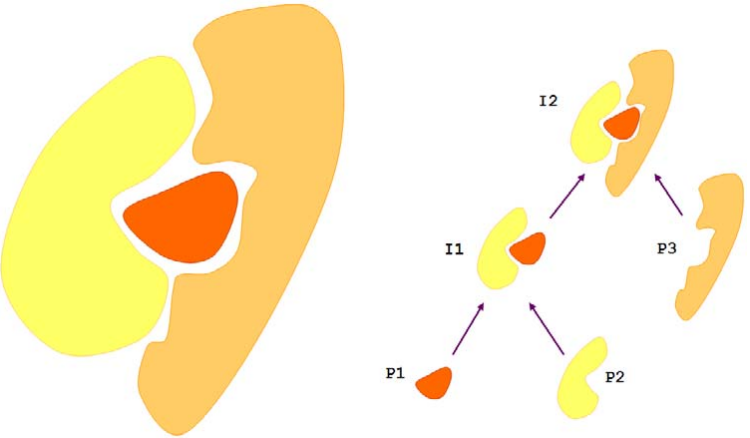
**Assembly of a mature complex (I2) from two protein components (P1 and P2) that bind to form an intermediate assembly (I1) with which a third protein (P3) interacts**. I1 is both the result of an interaction and a subsequent interactor in the PSI-MI2.5 format.

An *interactor *element describes a molecule participating in an *interaction*. In version 1.0, the corresponding element was named *proteinInteractor*, as version 1.0 was only intended to represent protein interactions. The original plan was to add additional interactor types, e.g. *rnaInteractor*, as separate elements in future versions of the schema, however, it quickly became apparent that a full modelling of all relevant interactor types, for example mRNA, tRNA, rRNA and so on, would make the schema unnecessarily repetitive and complex. Thus, version 2.5 contains a lightweight representation of an interacting molecule, with the key element *interactorType*, which qualifies an interactor with a term from the PSI-MI controlled vocabulary, for example 'protein' (MI:0326; see Availability and requirements for instructions on how to access this and other entries) or 'small nucleolar rna' (MI:0607) (Figure 2). This allows a compact, yet very expressive representation of an interactor. The schema does not attempt to provide a detailed representation of interacting molecules – *interactor *elements will usually contain only basic information and refer to external databases for detailed descriptions. The controlled vocabulary for *interactorType *currently comprises >25 terms and can be easily expanded should a user wish to describe an additional *interactor *type, for example phospholipids, without any change to the XML schema. Additional cross-references to external resources allow for a more detailed description of the interactor, for example to UniProtKB (MI:0018) [17] to further describe a protein molecule or ChEBI (MI:0474) [18] for a chemical entity (Figure 3). The schema also now supports the hierarchical build-up of complexes from component sub-complexes, in that a sub-complex can be recycled as an *interactor *using the appropriate controlled vocabulary term and internal referencing (Figure 4). This allows the visualisation of a series of consecutive events, for example in the case where a receptor must form from two or more subunits before its agonist can bind.

The *interaction *element describes one interaction. Within the *interaction *element, the *experimentList *either refers to or describes all experiments in which this interaction has been determined. The *participantList *lists all the molecules participating in an interaction. In the PSI-MI schema, an *interaction *can have one (e.g. in an autophosphorylation), two (e.g. in a yeast two-hybrid experiment) or multiple (e.g. in a tandem affinity purification (MI:0676) experiment) participants. While an *interactor *is the molecule in its 'canonical form' as represented in an external database, a *participant *element describes the specific instance of the interactor in the interaction. The *participant *must refer to an underlying *interactor*. In version 2.5, the *role *element of *participant *has been split into a *biologicalRole*, such as 'enzyme' (MI:0501), 'enzyme target' (MI:502) or 'cofactor' (MI:0682), and an *experimentalRole*, for example 'bait' (MI:0496) or 'prey' (MI:0498) (Figures 3 and 4). In addition, it is now possible to include multiple *experimentalRole*s for a given *participant*, each referring to one or more experiments in which the participant had this role. This can be used when an interaction in a yeast two-hybrid (MI:0018) experiment has been observed twice, with the participant bait/prey roles being reversed in the repeat experiment. The *experimentalPreparationList *allows the provision of terms from a controlled vocabulary to describe key aspects of the experimental preparation of each participant prior to the detection of the interaction, for example a protein might be an '*in vitro *translated protein' (MI:0589).

The method used for participant identification was described only at the experiment level in version 1.0. Whilst this is sufficient in most cases, it has been shown to be a limitation for experiments where molecules have been identified by different methods, for example in the case of an'antibait coimmunoprecipitation' (MI:0006) where some of the prey proteins could be identified by 'western blot' (MI:0113), and additional interacting molecules subsequently identified by 'mass spectrometry' (MI:0427). In PSI-MI XML2.5, the *participant *element contains a *participantIdentificationMethodList *where this information can be mapped.

Experimental constraints often require researchers to determine interactions in model systems. As an example, an experiment might be performed using mouse proteins, but the aim of the experiment is to make a statement about the homologous interaction (interolog) in human. The *experimentalInteractor *element allows the reporting of this fact. The *experimentalInteractor *will point to the model interactor used, while the human protein is referred to by the mandatory '*interactorRef' *element of the *participant*. Interactions of this type are referred to as 'modelled' interactions, and additionally flagged by the *modelled *element on the interaction level.

The *featureList *element describes the sequence features of the *participant *that are relevant for the interaction, using the appropriate term from the corresponding controlled vocabulary, for example 'DNA binding domains' (MI:0688) or experimental modifications such as 'biotin tag' (MI:0239). Features are classified by a controlled vocabulary linked from the *featureType *element. The *featureRangeList *describes the location of a feature on the sequence. This element has undergone extensive remodelling in version 2.5 to allow the representation of discontinuous sequence features and features of undetermined or general location such as 'n-terminal' (MI:0340).

The *negative *flag of an *interaction *is set to *true *if a particular interaction has been explicitly shown not to occur under the described conditions. Although it is optional (default value is *false*), it is obviously essential to correctly interpret this element when using PSI-MI formatted datasets.

The *confidenceList *provides an estimate of the confidence that this interaction actually occurs. The development of method-specific and method-independent measures for interaction confidence measures is an ongoing research topic, and thus the *confidenceList *allows the provision of multiple confidence measures for an interaction, usually based on different methods. A typical case would be one confidence measure provided by the original publication, and a separate confidence measure implemented by a public database.

In version 1.0 of the PSI-MI schema, the modelling of quantitative parameters was knowingly neglected, as very little quantitative data on protein interactions was available from public databases. In the current version, the *parameterList *on both *interaction *and *participant *level allows the flexible addition of kinetic parameters, which are becoming more available now through technologies like 'surface plasmon resonance' (MI:0107), and are being systematically collected by many resources, for example the BindingDB database [19].

The major XML schema elements contain a set of recurring standard elements, listed below.

(1) The *id *attribute provides an identifier to the object that must be unique to that object within the PSI-MI file. An object can be repeated, however, if the extended form of the schema is used. The *id *object is not necessarily stable, stable identifiers are provided in the *xref *element, see below.

(2) The *names *element to provide short labels (LCK_HUMAN), fully detailed names (Proto-oncogene tyrosine-protein kinase LCK), and synonyms (p56-LCK).

(3) The *xref *element to reference external database entities. Each *xref *element contains a single primary and optionally multiple secondary references that enable the referencing of both a primary data source, and a related secondary source such as a method publication. The specific reference type can be provided as an attribute *refType*, which is itself defined by a controlled vocabulary. Learning from experience gained in implementing version 1.0, database cross-references now use the extended PSI-MI CV2.5 controlled vocabulary to ensure consistent naming of external database resources, avoiding problems with the unification of, for example, 'Swiss-Prot', 'swissprot', 'sp', 'uniprot' and 'uniprotkb'. This resource is now identified by the attribute *db *and the optional attribute *dbAc*, which contains the accession number for the database in the PSI-MI controlled vocabulary.

(4) The *attributeList *element provides the option of flexibly adding content to PSI-MI elements. Each attribute has a name derived from a controlled vocabulary, and a free text value. As the attribute *name *must be selected from the PSI-MI controlled vocabulary, the *attributeList *contents remain semi-structured, and allows easy searching for specific topics.

### Controlled vocabularies

**Figure 5:**
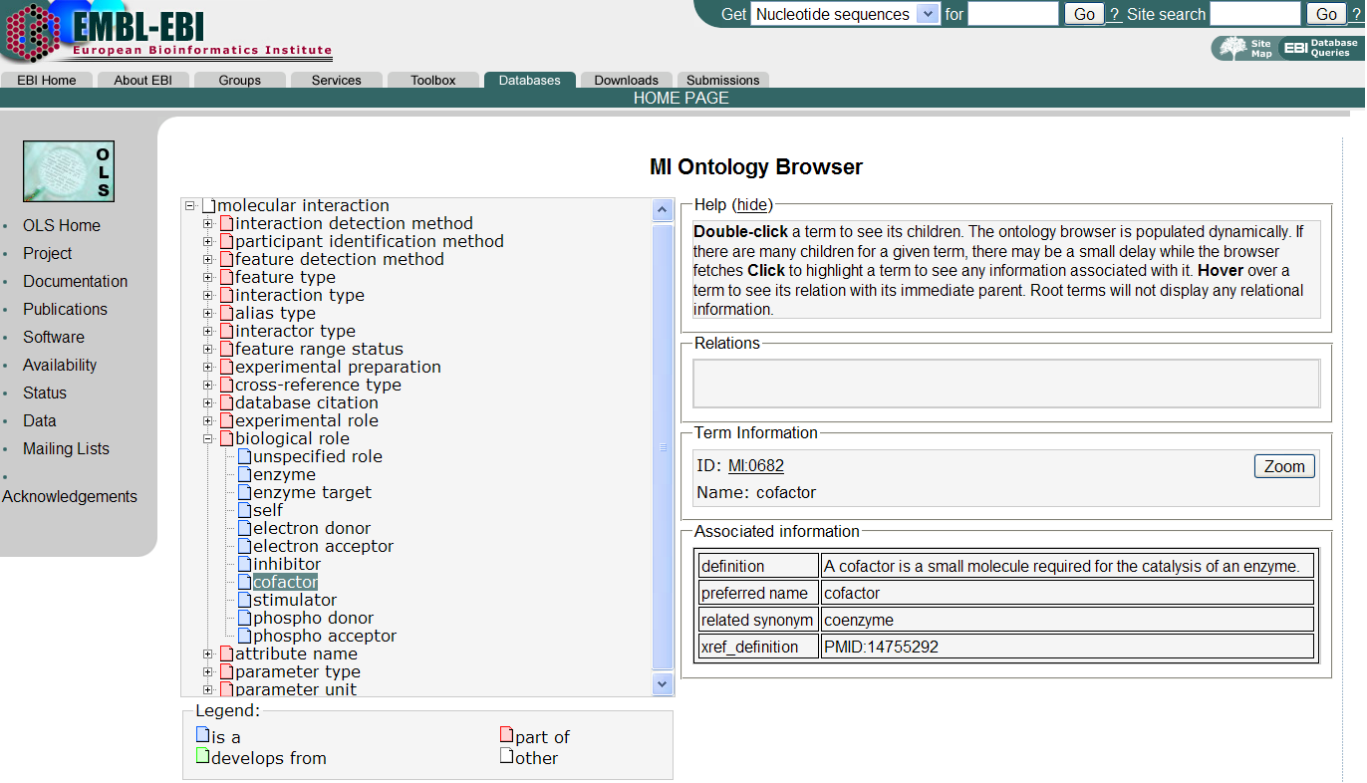
A representative section of the PSI-MI controlled vocabulary displayed in the Ontology Lookup Service.

Controlled vocabularies (CVs) are used throughout the PSI-MI schema to standardize the meaning of data objects. Their use ensures that the same term used throughout a description by a data producer, instead of a synonym or alternative spelling, and also that the interpretation of the meaning of that term remains consistent between multiple data producers and data users. In order to achieve this, all terms have definitions and, where appropriate, are supported by a literature reference. The controlled vocabularies have a hierarchical structure, higher level terms being more general than lower level descriptors, allowing annotation to be performed to an appropriate level of granularity whilst also enabling search tools to return all mapped objects to both parent and child terms, if required (Figure 5).

The descriptive ability of the PSI-MI controlled vocabularies has increased dramatically since their original release concurrent with PSI-MI version 1.0. The number of root terms has increased from 5 to 16 and the depth of each CV has been augmented by the addition of both new terms and the improved definitions of existing term. For example, on the release of PSI-MI 1.0, only a single term 'aggregation' was listed in the controlled vocabulary with the root 'interaction type'. This term proved to be too ambiguous to adequately describe the interaction annotated in major interaction databases, and unpopular with users, so it was made obsolete. The term 'interaction type' (MI:0190) now has three top-level terms 'genetic interaction' (MI:0208), 'physical interaction' (MI:0218) and 'colocalization' (MI:0403) all of which have numerous children. This allows an interaction to be described as, for example, an 'enzymatic reaction' (MI:0414) or even for the annotator to be as specific as 'dna strand elongation' (MI:0701). Expansion of the CVs has resulted in these replacing elements in the PSI-MI XML1.0 that were previously Boolean such as *isOverexpressedProtein *and *isTaggedProtein*. This now allows a much richer description of each term, such as the ability to state precisely which tag has been used and whether it was placed at the C-terminus or N-terminus of the molecule.

The CVs are maintained in the Open Biomedical Ontologies (OBO) [12] flat file format (psi-mi25.obo) with the namespace prefix 'MI' and are available both on the PSI-MI and the OBO websites or can be browsed using the Ontology Lookup Service (see below). The CVs are designed and actively developed to rapidly respond to changes in technology and methodology. This process is overseen by an elected editorial board and users can request new terms via a tracker on the PSI-MI website. We aim at responding to user requests within two weeks.

As backward compatibility between the PSI-MI XML1.0 and 2.5 was not fully achievable, given the support for new 2.5 features, a final version of the 1.0 CVs were frozen and are available from the PSI-MI website. A mapping file from CV2.5 to CV1.0 has been supplied on the PSI-MI web pages to enable providers to release data in 1.0 format but there will inevitably be data loss with reverse mapping and it is envisaged that this will increase as the CV2.5 further develop, so migration to 2.5 is recommended at the earliest possible opportunity.

### Tools

The development and maintenance of tools to enable the use of this format by a wide number of users is a community effort to which many people have contributed. All tools are freely available and can be accessed and downloaded from the PSI-MI web pages. These include applications to view and validate the use of the schema, to enable graphical representation of interaction network, to convert between data formats and to facilitate the use of controlled vocabularies. All tools are specific to the PSI-MI XML format apart from the validator.

### PSI-MI XML view

XML is a powerful means by which to model complex data while preserving human readability. However, due to the complexity of the PSI-MI schema we felt it important to provide an easier way to visualise the data. XSLT scripts have been made available in order to convert XML data files to HTML, thus providing user-friendly representation of the data.

### Conversion between expanded and compact form of the schema

A set of XSLT scripts has been made available to the community, thus allowing users to convert PSI-MI XML2.5 between compact and expanded forms.

### Validator

The PSI-MI data model representation is based on an XML Schema that represents how the data should be formatted. Schemas provide the means to define cardinality of XML elements and give type to their attributes but do not allow the enforcement of complex semantic rules. In order to overcome this limitation, a generic validation framework was created and a specific extension developed for the PSI-MI XML2.5 format. It allows the definition of very detailed rules such as checking that the biological role of participants involved in a yeast two-hybrid (MI:0018) experiment is either bait (MI:0496) or prey (MI:0498). As illustrated in the example above, the framework provides ways to interface with OBO format ontologies. Several clients currently allow the validation of PSI-MI XML2.5 data. These include a Java API that enables the embedding of the validator into any third party application (this is the most versatile application), a command line interface in addition to a graphical interface bundled with the Java API, and a web application that allows the uploading of a PSI-MI data file and reporting of both syntactic and semantic discrepancies [13] (after validation, it automatically creates the HTML view described above).

### Ontology Lookup Service (OLS)

Many ontologies are made available using the OBO format. OLS is an AJAX-based ontology viewer that also acts as a portal to access all the controlled vocabularies currently maintained on the Open Biomedical Ontologies website, enabling the searching of the PSI-MI controlled vocabulary by term name, id or synonyms [20]. Auto-completion provides a user-friendly search mechanism. A programmatic interface allows the embedding of OLS into any SOAP enabled application.

### Java XML parser

Many molecular interaction databases have chosen the PSI-MI format for providing data to their users. In order to ease the development of tools exploiting this data, a Java library providing a user friendly data model and the core functionalities for reading and writing PSI-MI XML2.5 data. Furthermore the parser supports XML streaming, thus providing a versatile and cursor based approach for retrieving interactions, interactors or experiments. The parser has been made available on the PSI-MI website.

### XMLMaker/Flattener

The XMLMaker/Flattener is a Java application that converts any XML schema into tab-delimited ASCII format (flat-files) and vice versa, given a user-defined mapping that can be saved and reused on subsequent files. A PSI-MI mapping can be readily created to inter-convert PSI-MI 1.0 or 2.5 XML files to simple flat files.

We recognize that some of the tools listed above have limitations with respect to memory requirements when dealing with large data files and expanded/compact forms of the schema. The latter problem can be addressed by choosing the appropriate XSLT script.

### Relationship with other community resources

The PSI-MI standard is only one of several inter-related efforts of the proteomics community to develop standards for data and XML-interchange formats [14]. These standards have been developed as part of the broader standards community and care is being taken to avoid overlaps, such that if a process is described in one standard it need only be referred to from others employing the same technology. For example, the controlled vocabularies used throughout all of the PSI-XML schemas are mapped to the schema, rather than being embedded within them to enable use of other useful CVs as they arise. For example, should a particular mass spectrometry technique be used to identify a participant, the appropriate term can be taken from the PSI-Mass Spectrometry CV rather than separately listed within the PSI-MI CVs.

### BioPAX

The Biological Pathway Exchange (BioPAX) format is a collaboratively developed data exchange format for biological pathway data [15], that currently uses the PSI-MI ontology internally for modelling associated molecular interactions [16]. All PSI-MI entries annotated to 'physical interaction' map to the BioPAX *physicalInteraction *class.

### Cytoscape

Cytoscape is a powerful open-source resource for analysing and visualizing biological networks [21]. The Cytoscape user community has developed numerous plugins allowing the extension of its functionalities in the area of data format compatibility and network analysis. Cytoscape now allows users to load molecular interaction data in PSI-MI XML1.0 and 2.5 formats without installing additional extensions, enabling data retrieval from one or more databases and its subsequent integration with data from other sources such as high-throughput expression experiments.

### The tabular form: MITAB2.5

The PSI-MI XML2.5 format allows a detailed representation of fully annotated interaction records both for inter-database and database-end user data transmission. However, to support many use cases, such as fast Perl parsing or loading into Microsoft Excel, that only require a simple, tabular format of interaction records, the MITAB2.5 format was defined as part of PSI-MI 2.5. The MITAB2.5 format (Additional files 1, 2, 3, 4, 5) has been derived from the tabular format provided by BioGRID [22]. MITAB2.5 only describes binary interactions, one pair of interactors per row. Columns are separated by tabulators and the contents should be as follows:

1. Unique identifier for interactor A, represented as databaseName:ac, where databaseName is the name of the corresponding database as defined in the PSI-MI controlled vocabulary, and ac is the unique primary identifier of the molecule in the database. Identifiers from multiple databases can be separated by '|'. It is recommended that proteins be identified using stable identifiers such as their UniProtKB or RefSeq [24] accession number.

2. Unique identifier for interactor B.

3. Alternative identifier for interactor A, for example the official gene symbol as defined by a recognised nomenclature committee. Representation as databaseName:identifier. Multiple identifiers separated by '|'.

4. Alternative identifier for interactor B.

5. Aliases for A, separated by '|'. Representation as databaseName:identifier. Multiple identifiers separated by '|'.

6. Aliases for B.

7. Interaction detection methods, taken from the corresponding PSI-MI controlled vocabulary, and represented as databaseName:identifier (methodName), separated by '|'.

8. First author surname(s) of the publication(s) in which this interaction has been shown, optionally followed by additional indicators, e.g. 'Smith-2005-a'. Separated by '|'.

9. Identifier of the publication in which this interaction has been shown. Database name, usually PubMed (MI:0446), taken from the PSI-MI controlled vocabulary, represented as databaseName:identifier. Multiple identifiers separated by '|'.

10. NCBI Taxonomy identifier for interactor A. Database name for NCBI taxid taken from the PSI-MI controlled vocabulary, represented as databaseName:identifier. Multiple identifiers separated by '|'. Note that in this column, the databaseName:identifier(speciesName) notation is only there for consistency. Currently no taxonomy identifiers other than NCBI taxid are anticipated, apart from the use of -1 to indicate '*in vitro*', -2 to indicate 'chemical synthesis', -3 to indicate 'unknown', -4 to indicate '*in vivo*' and -5 to indicate '*in silico*'.

11. NCBI Taxonomy identifier for interactor B.

12. Interaction types, taken from the corresponding PSI-MI controlled vocabulary, and represented as dataBaseName:identifier(interactionType), separated by '|'.

13. Source databases and identifiers, taken from the corresponding PSI-MI controlled vocabulary, and represented as databaseName:identifier(sourceName). Multiple source databases can be separated by '|'.

14. Interaction identifier(s) in the corresponding source database, represented by databaseName:identifier.

15. Confidence score. Denoted as scoreType:value. There are many different types of confidence score, but so far no controlled vocabulary. Thus the only current recommendation is to use score types consistently within one source. Multiple scores separated by '|'.

16. Allfurther columns are currently undefined.

All columns are mandatory. Missing values (empty cells) are marked by '-'.

### MITAB2.5 API

In order to ease the conversion from PSI-MI XML2.5 to MITAB2.5, a Java API was developed so data collected from various data providers can be easily integrated. A user-friendly graphical interface allows the user to drag and drop a set of PSI-MI XML2.5 files and convert them into one (or many) MITAB2.5 file(s). Additionally, whenever interactions involving more than two participants, for example many coimmunoprecipitation experiments, are encountered, either Spoke or Matrix expansion can be applied to the data in order to result in only binary interactions.

### Data availability

Interaction data is available in PSI-MI XML2.5 format from DIP, IntAct, MINT, MIPS, BioGRID and the HPRD databases. MINT, IntAct and DIP also supply data in MITAB2.5 format. Building on the PSI-MI standard, major public interaction databases have formed the International Molecular Interaction Exchange consortium (IMEx) [23]. These databases, currently DIP, IntAct, MINT and MPact (MIPS), have started to share the curation load and aim to regularly interchange data curated to the same common standards, in a manner similar to the well-established pattern followed by the nucleotide sequence databases. IMEx downloads are currently available from the DIP, IntAct and MINT FTP sites.

### Submissions

A number of databases actively encourage direct submissions by authors. Members of the IMEx Consortium host a number of tools and resources to assist in this process [25]. Any data submitted via this website will be exchanged between participating databases and made available, on publication of the accompanying manuscript, from all database websites in both PSI-MI XML2.5 and MITAB2.5 formats.

### Sample entry

Sample data throughout this paper have been taken from [26] (see also Availability and requirements). The full data set can be viewed as supplementary materials in both PSI-MI 2.5 XML or MITAB2.5 formats or can be viewed as a fully curated dataset in the IntAct database (EBI-914159, EBI-914232, EBI-914360).

## Conclusion

The PSI-MI XML1.0 format provided the molecular interaction community with a common mechanism for data exchange that enabled data to be shared between multiple sources, integrated, analysed and visualised by a range of software tools. Its rapid and widespread acceptance showed that this was a much needed resource, but it also soon became apparent that version 1.0 was limited in scope and that the community required a broader, more flexible, format. We have presented PSI-MI XML2.5, with the increased expressive power, which allows more detailed and complete representation of interaction data, including the ability to express interactions involving any interactor type. The accompanying controlled vocabularies have been expanded and improved to further enable detailed annotation of interaction data. Tool development has continued in parallel and a suite of programming resources now exist to assist the user in producing and manipulating valid PSI-MI XML2.5 and MITAB2.5 files.

The original stated intention was to develop PSI-MI in a multi-levelled approach, with each level being backward compatible with earlier versions. Due to the complexity of interaction data already available in the public domain, this aim proved impossible to achieve, with a rewrite of version 1.0 proving necessary to enable all this information to be expressed within the interchange format. The restructuring of the XML format required a concomitant major reorganization of the controlled vocabulary. Version 1.0, and the accompanying CVs, has therefore been frozen and is maintained in support of users who have already started to use this format, but it is recommended that all users switch to the more versatile version 2.5 as soon as possible. Whilst the controlled vocabulary will continue to quickly adopt to the needs of the community, the PSI-MI XML2.5 format is expected to stay stable at least for two to three years, as with version 1.0, which was published in early 2004.

The benefits of PSI-MI adoption are clear, including better cooperation between public domain databases, culminating in the formation of the IMEx consortium to share the load of collecting vast amounts of interactions from the literature. Scientists can now download and combine the contents of multiple databases to populate a local resource using a single data format, enabling easy comparison with experimental results generated in their own laboratory.

There is still much work to be undertaken in further developing both the controlled vocabularies and accompanying tools. A PSI common query interface API for all participating databases, PSICQUIC is under development. Work is also in hand to investigate use of the current format to enable the use of the PSI-MI XML2.5 format for transferring information on antibody properties such as specificity, cross-reactivity, affinity and avidity. This will require extensions to many of the current controlled vocabularies such as those describing interactors and also extending the current kinetic parameters.

The PSI effort requires support from the user community to continue to expand, thus is actively seeking input and advice from all quarters. Anyone wishing to become involved is invited to visit the PSI website [14], to participate in the discussion groups listed, and to contribute to the further development of community standards for proteomics data.

## Availability and requirements

The PSI XML2.5 schema, documentation, and tools are freely available from the PSI MI website [8]. The entries throughout the text can be accessed by appending them to the root URL  where xx is the two letter code before the colon and XXXX is the four digit code after the colon.

## Competing interests

The author(s) declares that there are no competing interests.

## Authors' contributions

SK, SO contributed to the schema development, controlled vocabulary development, the tools development and the writing of the manuscript. LM-P, AC, MO, VS, LS, JN contributed to the schema development, controlled vocabulary development and the tools. BA, AFQ, NV, GDB, EC contributed to the tool development. IX, JW, DS, JJS, SM, MEC, JA, PW, AC-A contributed to the schema development and the controlled vocabulary development. MT, MG contributed to the schema development. HH, MV, CH, DE, GC, RA conceived of the concept. All authors read and approved the final manuscript.

## Supplementary Material

Additional file 1 **PSI-MI XML2.5 example**. Data from [26] represented in PSI-MI XML2.5 format in compact form. Click here for file

Additional file 2 **PSI-MI XML2.5 example in HTML**. Data from [26] represented in PSI-MI XML2.5 format in compact form, converted to HTML. Click here for file

Additional file 3 **PSI-MI XML2.5 example**. Data from [26] represented in PSI-MI XML2.5 format in expanded form. Click here for file

Additional file 4 **PSI-MI XML2.5 example in HTML**. Data from [26] represented in PSI-MI XML2.5 format in expanded form, converted to HTML. Click here for file

Additional file 5 **MITAB2.5 example**. Data from [26] represented in MITAB2.5 format. Click here for file
