# Supplementary material for: Broadening the horizon – level 2.5 of the HUPO-PSI format for molecular interactions
Source: BMC Biol. 2007 Oct 9;5:44. doi: 10.1186/1741-7007-5-44 (PMC2189715; doi:10.1186/1741-7007-5-44)
Supplement: Additional file 2 — PSI-MI XML2.5 example in HTML. Data from [26] represented in PSI-MI XML2.5 format in compact form, converted to HTML. [file 1741-7007-5-44-S2.html]

HUPO Proteomics Standards Initiative
Molecular Interaction


## Proteomics Standards Initiative

## Molecular Interaction Version 2.5

|  |  |
| --- | --- |
| Source | |
| Name: | European Bioinformatics Institute |
| pubmed | 14681455 |
| psi-mi | MI:0469 |
| postalAddress | Wellcome Trust Genome Campus, Hinxton, Cambridge, CB10 1SD, United Kingdom |
| url | http://www.ebi.ac.uk |


|  |  |
| --- | --- |
| AvailabilityList | |


|  |  |
| --- | --- |
| ExperimentList | |
| Experiment #2 | |
| Name: | hobson-2001-1 |
| Description: | Oligomeric structures of poliovirus polymerase are important for function. |
| pubmed | 11230138 |
| intact | EBI-914159 (hobson-2001-1) |
| Host Organism: | in vitro |
| Interaction detection method: | |  |  | | --- | --- | | Name: | filter binding (Filter overlay assay) | | psi-mi | MI:0049 | | pubmed | 7708014 | |
| Participant identification method: | |  |  | | --- | --- | | Name: | predetermined: predetermined participant | | psi-mi | MI:0396 | | pubmed | 7940758 | | pubmed | 14755292 | |
|
| author-list | Hobson SD., Rosenblum ES., Richards OC., Richmond K., Kirkegaard K., Schultz SC. |
| contact-email | scott.hobson@cellzome.de |
| publication-year | 2001 |
| exp-modification | RNA binding was evaluated using a nitrocellulose filter binding assay. The amount of radiolabelled RNA bound to each membrane was quantified using a PhosphorImager |
| journal | EMBO J. (0261-4189) |
| data-processing | Residues renumbered to match full-length polyprotein for this virus. |
| Experiment #25 | |
| Name: | hobson-2001-2 |
| Description: | Oligomeric structures of poliovirus polymerase are important for function. |
| pubmed | 11230138 |
| intact | EBI-914232 (hobson-2001-2) |
| Host Organism: | in vitro |
| Interaction detection method: | |  |  | | --- | --- | | Name: | rna rna pol assay: rna directed rna polymerase assay | | psi-mi | MI:0700 | | pubmed | 14755292 | |
| Participant identification method: | |  |  | | --- | --- | | Name: | predetermined: predetermined participant | | psi-mi | MI:0396 | | pubmed | 7940758 | | pubmed | 14755292 | |
|
| author-list | Hobson SD., Rosenblum ES., Richards OC., Richmond K., Kirkegaard K., Schultz SC. |
| contact-email | scott.hobson@cellzome.de |
| publication-year | 2001 |
| exp-modification | Elongation assays evaluated using radiolabelled RNA as a substrate. Elongated products were separated by PAGE and visualised using a PhosphoImager |
| journal | EMBO J. (0261-4189) |
| data-processing | Residues renumbered to match full-length polyprotein for this virus. |
| Experiment #41 | |
| Name: | hobson-2001-3 |
| Description: | Oligomeric structures of poliovirus polymerase are important for function. |
| pubmed | 11230138 |
| intact | EBI-914360 (hobson-2001-3) |
| Host Organism: | in vitro |
| Interaction detection method: | |  |  | | --- | --- | | Name: | crosslink: cross-linking study | | psi-mi | MI:0030 | | pubmed | 14755292 | |
| Participant identification method: | |  |  | | --- | --- | | Name: | predetermined: predetermined participant | | psi-mi | MI:0396 | | pubmed | 7940758 | | pubmed | 14755292 | |
|
| author-list | Hobson SD., Rosenblum ES., Richards OC., Richmond K., Kirkegaard K., Schultz SC. |
| contact-email | scott.hobson@cellzome.de |
| publication-year | 2001 |
| journal | EMBO J. (0261-4189) |
| exp-modification | 2mM dithiothreotol added to reaction mixture, this was increased to 50mM for reducing conditions. Proteins seperated by SDS-PAGE and visualised by Coomassie staining |
| data-processing | Residues renumbered to match full-length polyprotein for this virus. |


|  |  |
| --- | --- |
| InteractorList | |
| Interactor #12 | |
| Name: | ggcagcugcaugcagcugcc: 5' -3' RNA synthesized for use in PMID: 11230138 |
| intact | EBI-914168 (ggcagcugcaugcagcugcc) |
| Interactor Type: | |  |  | | --- | --- | | Name: | rna: ribonucleic acid (RNA) | | psi-mi | MI:0320 | | pubmed | 14755292 | | so | SO:0000356 | |
| Organism: | in vitro |
| Sequence: | GGCAGCUGCA UGCAGCUGCC |
| Interactor #15 | |
| Name: | ccgucgacguacgucgac-2: 3'-5' RNA strand synthesised for use in PMID: 11230138 |
| intact | EBI-944576 (ccgucgacguacgucgac-2) |
| Interactor Type: | |  |  | | --- | --- | | Name: | rna: ribonucleic acid (RNA) | | psi-mi | MI:0320 | | pubmed | 14755292 | | so | SO:0000356 | |
| Organism: | in vitro |
| Sequence: | CGUCGACGUA CGUCGACGGC GCGG |
| Interactor #23 | |
| Name: | ggcgcggcagcugcaugcag: 5'-3' RNA synthesized for use in PMID: 11230138 |
| intact | EBI-914181 (ggcgcggcagcugcaugcag) |
| Interactor Type: | |  |  | | --- | --- | | Name: | rna: ribonucleic acid (RNA) | | psi-mi | MI:0320 | | pubmed | 14755292 | | so | SO:0000356 | |
| Organism: | in vitro |
| Sequence: | GGCGCGGCAG CUGCAUGCAG CUGCC |
| Interactor #27 | |
| Name: | zinc 2plus: zinc(2+) |
| chebi | CHEBI:29105 |
| intact | EBI-968340 (zinc 2plus) |
| Interactor Type: | |  |  | | --- | --- | | Name: | small molecule | | psi-mi | MI:0328 | | pubmed | 14755292 | |
| Interactor #4 | |
| Name: | ccgucgacguacgucgacgg: 3' - 5' RNA strand synthesised for use in PMID: 11230138 |
| intact | EBI-944574 (ccgucgacguacgucgacgg) |
| Interactor Type: | |  |  | | --- | --- | | Name: | rna: ribonucleic acid (RNA) | | psi-mi | MI:0320 | | pubmed | 14755292 | | so | SO:0000356 | |
| Organism: | in vitro |
| Sequence: | CCGUCGACGU ACGUCGACGG |
| Interactor #6 | |
| Name: | p03300-pro\_000004009: Poliovirus type 1 (strain Mahoney) RNA-directed RNA polymerase |
| uniprotkb | P03300-PRO\_0000040090 |
| refseq | NP\_740478.2 |
| intact | EBI-914162 (p03300-pro\_000004009) |
| Interactor Type: | |  |  | | --- | --- | | Name: | peptide (oligopeptide) (polypeptide) | | psi-mi | MI:0327 | | pubmed | 14755292 | |
| Organism: | pol1m: Poliovirus type 1 (strain Mahoney) |
| no-uniprot-update | An amino acid sequence which is not represented in UniProt |


|  |  |
| --- | --- |
| InteractionList | |
| Interaction #1 | |
| Name: | polg-rna-1: Poliovirus type 1 (strain Mahoney) binds double stranded RNA |
| intact | EBI-914164 (polg-rna-1) |
| go | GO:0003725 (double-stranded RNA binding) |
| Experiments: | hobson-2001-1 |
| Participant #3 | |  |  | | --- | --- | | ccgucgacguacgucgacgg | | | Biological Role: | |  |  | | --- | --- | | Name: | unspecified role | | psi-mi | MI:0499 | | pubmed | 14755292 | | | Experimental Role: | |  |  | | --- | --- | | Name: | prey | | psi-mi | MI:0498 | | pubmed | 14755292 | | |
| Participant #5 | |  |  | | --- | --- | | p03300-pro\_000004009 | | | Biological Role: | |  |  | | --- | --- | | Name: | unspecified role | | psi-mi | MI:0499 | | pubmed | 14755292 | | | Experimental Role: | |  |  | | --- | --- | | Name: | bait | | psi-mi | MI:0496 | | pubmed | 14755292 | | | Feature #7 | mutation decreasing (leu2089ala) [ 2089 .. 2089 ] | | Feature #8 | mutation decreasing (asp2096arg) [ 2096 .. 2096 ] | | Feature #9 | mutation decreasing (arg2202asp) [ 2202 .. 2202 ] | | Feature #10 | mutation decreasing (arg2203asp) [ 2203 .. 2203 ] | | Host Organism: | pol1m: Poliovirus type 1 (strain Mahoney) | |
| Participant #11 | |  |  | | --- | --- | | ggcagcugcaugcagcugcc | | | Biological Role: | |  |  | | --- | --- | | Name: | unspecified role | | psi-mi | MI:0499 | | pubmed | 14755292 | | | Experimental Role: | |  |  | | --- | --- | | Name: | prey | | psi-mi | MI:0498 | | pubmed | 14755292 | | |
 Interaction Type: | |  |  | | --- | --- | | Name: | direct interaction | | psi-mi | MI:0407 | | pubmed | 14755292 | || kinetics | ED50 (wild-type)=3x10exp-6M |
| comment | RNA strands bind to form a blunt ended double strand, authors name 0.20 RNA |
| figure legend | 2A, 2C |
| comment | Protein bound single stranded RNA's with approximately 10 fold lower affinity. |
| kd | 0.0 |
| Interaction #13 | |
| Name: | polg-rna-2: Poliovirus type 1 (strain Mahoney) binds double stranded RNA |
| intact | EBI-914187 (polg-rna-2) |
| go | GO:0003725 (double-stranded RNA binding) |
| Experiments: | hobson-2001-1 |
| Participant #14 | |  |  | | --- | --- | | ccgucgacguacgucgac-2 | | | Biological Role: | |  |  | | --- | --- | | Name: | unspecified role | | psi-mi | MI:0499 | | pubmed | 14755292 | | | Experimental Role: | |  |  | | --- | --- | | Name: | prey | | psi-mi | MI:0498 | | pubmed | 14755292 | | |
| Participant #16 | |  |  | | --- | --- | | p03300-pro\_000004009 | | | Biological Role: | |  |  | | --- | --- | | Name: | unspecified role | | psi-mi | MI:0499 | | pubmed | 14755292 | | | Experimental Role: | |  |  | | --- | --- | | Name: | bait | | psi-mi | MI:0496 | | pubmed | 14755292 | | | Feature #17 | sufficient to bind (region) [ 1812 .. 2208 ] | | Feature #18 | mutation decreasing (asp2096arg) [ 2096 .. 2096 ] | | Feature #19 | mutation decreasing (arg2202asp) [ 2202 .. 2202 ] | | Feature #20 | mutation decreasing (arg2203asp) [ 2203 .. 2203 ] | | Feature #21 | mutation decreasing (leu2089ala) [ 2089 .. 2089 ] | | Host Organism: | pol1m: Poliovirus type 1 (strain Mahoney) | |
| Participant #22 | |  |  | | --- | --- | | ggcgcggcagcugcaugcag | | | Biological Role: | |  |  | | --- | --- | | Name: | unspecified role | | psi-mi | MI:0499 | | pubmed | 14755292 | | | Experimental Role: | |  |  | | --- | --- | | Name: | prey | | psi-mi | MI:0498 | | pubmed | 14755292 | | |
 Interaction Type: | |  |  | | --- | --- | | Name: | direct interaction | | psi-mi | MI:0407 | | pubmed | 14755292 | || kinetics | ED50 (wild-type)=6x10exp-6M |
| comment | RNA strands bind to form a blunt ended double strand, authors name 5.20 RNA |
| figure legend | 2A, 2D |
| comment | Protein bound single stranded RNA's with approximately 10 fold lower affinity. |
| kd | 0.0 |
| Interaction #24 | |
| Name: | polg-rna-3: Poliovirus type 1 (strain Mahoney) binds double stranded RNA |
| intact | EBI-914242 (polg-rna-3) |
| go | GO:0003725 (double-stranded RNA binding) |
| go | GO:0003968 (RNA-directed RNA polymerase ac) |
| go | GO:0006354 (RNA elongation) |
| go | GO:0008270 (zinc ion binding) |
| Experiments: | hobson-2001-2 |
| Participant #26 | |  |  | | --- | --- | | zinc 2plus | | | Biological Role: | |  |  | | --- | --- | | Name: | cofactor (coenzyme) | | psi-mi | MI:0682 | | pubmed | 14755292 | | | Experimental Role: | |  |  | | --- | --- | | Name: | cofactor (coenzyme) | | psi-mi | MI:0682 | | pubmed | 14755292 | | |
| Participant #28 | |  |  | | --- | --- | | ccgucgacguacgucgac-2 | | | Biological Role: | |  |  | | --- | --- | | Name: | enzyme target (substrate) | | psi-mi | MI:0502 | | pubmed | 14755292 | | | Experimental Role: | |  |  | | --- | --- | | Name: | neutral component | | psi-mi | MI:0497 | | pubmed | 14755292 | | |
| Participant #29 | |  |  | | --- | --- | | p03300-pro\_000004009 | | | Biological Role: | |  |  | | --- | --- | | Name: | enzyme | | psi-mi | MI:0501 | | pubmed | 14755292 | | | Experimental Role: | |  |  | | --- | --- | | Name: | neutral component | | psi-mi | MI:0497 | | pubmed | 14755292 | | | Feature #30 | required to bind (region) [ 1747 .. 1812 ] | | Feature #31 | mutation decreasing (ala1776cys) [ 1776 .. 1776 ] | | Feature #32 | mutation decreasing (region) [ 1777 .. 1777 ] | | Feature #33 | mutation decreasing (iso2188cys) [ 2188 .. 2188 ] | | Feature #34 | mutation decreasing (gly2189leu) [ 2189 .. 2189 ] | | Feature #35 | mutation decreasing (asp2096arg) [ 2096 .. 2096 ] | | Feature #36 | mutation decreasing (arg2202asp) [ 2202 .. 2202 ] | | Feature #37 | mutation decreasing (arg2203asp) [ 2203 .. 2203 ] | | Feature #38 | mutation decreasing (leu2089ala) [ 2089 .. 2089 ] | | Host Organism: | pol1m: Poliovirus type 1 (strain Mahoney) | |
| Participant #39 | |  |  | | --- | --- | | ggcgcggcagcugcaugcag | | | Biological Role: | |  |  | | --- | --- | | Name: | enzyme target (substrate) | | psi-mi | MI:0502 | | pubmed | 14755292 | | | Experimental Role: | |  |  | | --- | --- | | Name: | neutral component | | psi-mi | MI:0497 | | pubmed | 14755292 | | |
 Interaction Type: | |  |  | | --- | --- | | Name: | elongation: dna strand elongation (DNA replication elongation) | | psi-mi | MI:0701 | | pubmed | 14755292 | | go | GO:0006271 | || figure legend | Figure 2F and Figure 3 |
| comment | RNA strands bind to form a blunt ended double stranded RNA, authors name 5.20 RNA |
| kd | 0.0 |
| Interaction #40 | |
| Name: | polg-polg-1: Dimerization of Poliovirus type 1 (strain Mahoney) |
| intact | EBI-944619 (polg-polg-1) |
| go | GO:0042803 (protein homodimerization activ) |
| Experiments: | hobson-2001-3 |
| Participant #42 | |  |  | | --- | --- | | p03300-pro\_000004009 | | | Biological Role: | |  |  | | --- | --- | | Name: | unspecified role | | psi-mi | MI:0499 | | pubmed | 14755292 | | | Experimental Role: | |  |  | | --- | --- | | Name: | neutral component | | psi-mi | MI:0497 | | pubmed | 14755292 | | | Feature #43 | mutation increasing (ala1776cys) [ 1776 .. 1776 ] | | Host Organism: | pol1m: Poliovirus type 1 (strain Mahoney) | |
| Participant #44 | |  |  | | --- | --- | | p03300-pro\_000004009 | | | Biological Role: | |  |  | | --- | --- | | Name: | unspecified role | | psi-mi | MI:0499 | | pubmed | 14755292 | | | Experimental Role: | |  |  | | --- | --- | | Name: | neutral component | | psi-mi | MI:0497 | | pubmed | 14755292 | | | Feature #45 | mutation increasing (iso2188cys) [ 2188 .. 2188 ] | | Host Organism: | pol1m: Poliovirus type 1 (strain Mahoney) | |
 Interaction Type: | |  |  | | --- | --- | | Name: | direct interaction | | psi-mi | MI:0407 | | pubmed | 14755292 | || figure legend | 6E |
| comment | Under reducing conditions, only protein monomers are observed. |
| kd | 0.0 |
